# Bones, shells and baselines—how the past can inform modern marine management, protection and restoration

**DOI:** 10.1098/rstb.2024.0043

**Published:** 2025-07-10

**Authors:** Callum M. Roberts, Ruth H. Thurstan, James Scourse

**Affiliations:** ^1^ Faculty of Environment Science and Economy, Centre for Ecology and Conservation, University of Exeter, Exeter, UK; ^2^ Centre for Ecology and Conservation, University of Exeter, Exeter, UK; ^3^ Faculty of Environment, Science and Economy, University of Exeter, Exeter, UK

**Keywords:** prehistory, historical ecology, environmental reconstruction, ocean conservation

## Abstract

In this article, we explore how archaeological and historical perspectives can inform current marine management, protection and restoration efforts, and entice us to rethink our present-day relationships with ocean resources to secure sustainable management. Historical records, archaeological findings, palaeoecological data, local and traditional ecological knowledge are coming together in highly interdisciplinary ways, combining cutting-edge analytical and traditional methods. This research is producing more detailed and nuanced descriptions of past ecosystems that reveal the nature, scale and timing of human influences on the sea in unprecedented detail. The findings help us reset ‘shifted baselines’—a phenomenon where long-altered ecosystems are perceived as natural. By bringing clarity to historical human impacts, we can challenge current management paradigms, raise ambitions for recovery of biodiversity loss, improve approaches to habitat restoration and identify promising targets for rewilding. A deeper appreciation of what vibrant seas really look like helps us value and better communicate the benefits recovery can bring for society. Understanding ocean responses to past climates helps us predict and respond to emerging climate pressures today, helping managers develop better approaches to enhance resilience in the face of ongoing and future environmental changes.

This article is part of the theme issue ‘Shifting seas: understanding deep-time human impacts on marine ecosystems’.

## 1. The value of understanding past ecosystem states

We are in the midst of exciting methodological advances that enable us to better understand past marine environments, piece together how people used them and unravel what impacts, if any, they had. The use of environmental DNA (eDNA), bulk and compound-specific stable isotopic analyses, environmental proxies, zooarchaeology and modelling, for example, enable us to reconstruct past ocean conditions, identify the creatures present in different time periods, explore the evolution of trophic structure and, in some cases, detect how organisms have altered in abundance over time. These methods are also beginning to resolve changing patterns of human use and exploitation. Deep time approaches undeniably hold great value in improving our understanding of past seas and human influences through time, but how do we best apply these scientific discoveries to contemporary ecological problems?

The value of understanding past marine ecosystems for present-day conservation and management, and the magnitude of human influences upon them, is slowly becoming recognized. In the highly human-impacted oceans of today, without deeper time perspectives it is impossible to know what well-functioning, vibrant ecosystems look like and are capable of. How many whales can an ocean sustain? What does a seabed look like that has not been scoured by fish trawls and shellfish dredges for decades or centuries?

Our continuing need for ocean resources, accelerating climate change and the cumulative effects of centuries of human impact upon marine ecosystems mean environmental managers can rarely turn back the clock and recreate the past. Ecosystems are shaped by their environment as well as people, and past conditions were different from today’s. Looking to the past for ‘absolute baselines’ against which ‘conservation progress’ might be measured can also be problematic. Human societal influence and past environmental conditions have always been in flux: societies have at times deeply degraded, maintained or accelerated marine ecosystem services and functions, while shifting environmental conditions—both human- and non-human influenced—have also driven ecosystem change.

Despite these inherent limitations, data from archaeological and historical perspectives offer far more for marine conservation and management than is typically recognized. Though still not widespread, recognition of the value of palaeoecological data for conservation and management practice is more mature for terrestrial than marine environments. This was advocated as long ago as the 1990s [1,2] and has been the subject of both expert [3] and user community reviews [4]; it has been explored most fully in the context of the imperative to conserve ecologically extremely sensitive island ecosystems [5].

Conservation and management have long been plagued by ‘shifted baselines’, whereby the ecosystems we are familiar with today have been so long altered by human use and abuse that their changed conditions are considered natural. This shifted perspective has significant and lasting implications for how we manage our oceans and how we decide what to conserve or recover. The North Sea’s Dogger Bank, for example, had been fished intensively for hundreds of years before being given protection under European nature conservation laws in 2012. By this time, it had lost many of its largest and most iconic species, like skates, halibut and sturgeon. Other species were greatly depleted, and the seabed stripped of most of its epifauna. Yet nature conservation authorities, unaware of this richer past, proposed initially to maintain it in this state. Historical reconstruction of the Dogger’s past state inspired a different vision [6], enabling conservation organizations to press successfully for stronger protection with a ban on bottom trawling implemented in 2022 [7]. European and Australian flat oysters were all but wiped out by centuries of intensive and destructive over exploitation, combined with epidemics of introduced diseases [8,9]. Their numbers have been so long suppressed that conservationists thought the species never built actual reefs, but instead lived scattered over the seabed at low densities. A recent deeper dive into history reveals that they were reef builders, offering an opportunity for a reset in conservation and restoration practice [9,10].

Analyses of the past offer benchmarks from different points in time, inform us of the consequences of particular activities and set the health of our current ecosystems within the context of past ecological and societal changes. They offer insights as to how past societies interacted with and managed ocean resources and their ecological outcomes. This article identifies emerging knowledge on past ecosystems, and highlights some of the applications for management and conservation. We may not be able to turn back the clock, but we argue that enhancing our understanding of the past can offer valuable lessons and insights as we attempt to mitigate the negative influences of past and present human impact, and as we navigate environmental change into the future.

## 2. Building an understanding of past environmental conditions

Wildlife and the ecosystems they form are shaped by the physical and biogeochemical environments in which they occur. Understanding environmental differences and their role is a longstanding goal of historical reconstruction. Temperature and precipitation are the most important climatic factors shaping terrestrial ecosystems. In the sea, a combination of temperature, salinity, oxygen concentration and nutrient availability are crucial, knowledge of which can help unravel oceanographic conditions. In coastal contexts, sea level rise and fall, principally driven by glaciation, must be added to the mix [11].

One of the great limitations in building back a picture of the history of the environment and people of a place is how well their remains are preserved. In some contexts, there is fabulous preservation and events can be resolved in great detail, almost annual resolution in some cases; in other places, there are only vague traces. Chronologies of changing conditions on land and ecological responses to them are extracted from ice cores, lake-bed sediments or tree rings, for example. In the sea, marine sediments represent a primary archive from which to read signals of past environments. The best time series can be obtained from areas of relatively rapid sediment accumulation in places with low natural and human disturbance. Lake sediments have proved valuable repositories for records of anadromous fishes, offering insights into the ocean environment. For example, nutrient enrichment associated with salmon carcasses in an Alaskan lake provided a proxy for abundance from which century-scale variation in ocean productivity could be inferred across 2200 years [12]. Records of fish scale deposition in the anoxic Santa Barbara basin offered a similar long-term perspective on sardine and anchovy population variability and their oceanic drivers (13).

Exciting advances are also emerging from the field of sclerochronology. Here, annually resolved time series of environmental conditions can be extracted from dated chronologies of shells using the methods of tree-ring dating applied to growth rings of long-lived animals. Butler *et al*. [11] pieced together a 1357 year picture of ocean environmental change for the north Icelandic shelf based on analyses of growth rings across multiple *Arctica islandica* clam shells. Such records are being pushed back in time through the Holocene based on dating more ancient shell remains using a novel application of amino acid racemization [14].

## 3. Can we tell from past remains whether animals were more abundant than today?

A central question that palaeoenvironmental science seeks to answer is how many fish did the ocean support in the past? Often, animal remains have accumulated at sites of processing or consumption. Shell middens are probably the most obvious present-day symbols of past seafood exploitation, the largest of which rise as low hills. For example, in southern Brazil, middens rise to 50 m and extend over thousands of square metres [15]. These great heaps appear, at face value, to demonstrate prolific resources. However, low rates of exploitation of more limited resources, sustained over thousands of years, could produce similar results. Separating these possibilities requires robust dating of midden layers to resolve accumulation rates, and thereby exploitation intensities. The tools for fine-scale dating are improving all the time [16,17]. Hausman & Meredith-Williams [18], for example, resolved seasonal patterns in a midden accumulation in Saudi Arabia from stable oxygen isotopes in shell growth rings to estimate a consumption rate of 400 shells per day.

We are on more secure ground inferring differences between past and present when the animals represented in archaeological contexts are rare or absent from the local environment today. For example, Rodrigues *et al*. [19] used DNA barcoding and collagen fingerprinting to identify remains of three right and three grey whales from Roman archaeological sites in the Mediterranean. Northern right whales are today restricted to the western Atlantic, while Atlantic grey whales are extinct. Such absences can also be relative, such as when animals in the past reached sizes no longer present.

Of course, the remains in archaeological contexts represent a small subset of the wildlife present at the time of exploitation. The resources people used were limited by accessibility, technology and preferences and the remains have been further filtered by differences in preservation. Sometimes, wildlife remains offer insights into maritime technologies allowing us to infer, for example, the existence of hook and line, tens of thousands of years before the first physical remains of such technologies. For example, shark and tuna bones found in East Timor’s Jerimalai cave date to 43 000 years ago, but the earliest fishhooks appeared there 16 000 to 23 000 years ago [20]. The hooks were made from shells that preserved poorly.

It seems from many historical descriptions as if fish and wildlife abundance was far greater than it is today. However, modern ecological models can indicate that the ocean could not support such abundances today. Differing explanations include the possibility that past descriptions are inaccurate, or they derive from sites of exceptional abundance for their time. In some cases, studies have evidenced significant and surprising scales of ecological change as a result of human activities, which are likely to have influenced ocean productivity as well as seabed structure, such as the collapse of once-abundant oyster ecosystems (e.g. [10,21]). In other cases, resolving the reasons for such differences remains a work in progress. Inter- and transdisciplinary studies, whereby multiple knowledge forms, methodological approaches and datasets are integrated, offer a route to reducing uncertainties or to resolving differences in interpretation. For example, Efford *et al.* [22] combined archaeology, historical ecology and Indigenous knowledge, which together enabled them to model the cumulative environmental and human impacts of colonization in Burrard Inlet, Canada.

## 4. When did significant, detectable, human influence on the ocean begin, what forms did it take and how has it developed over time?

Long and vigorous arguments stretched across decades about whether modern climate change is happening or has a human cause, revealing eloquently the difficulties in disentangling human influences in naturally variable systems. Human influences in the distant past would have been slight and localized, and therefore hard to detect, particularly based on the remains and proxies available to us now. The earliest use people made of the sea was to collect and catch seafood. Mollusc remains found in a South African cave midden represent some of the earliest recorded evidence of human consumption of seafood, dating to 164 000 years ago [23]. A near-universal characteristic of fisheries is to reduce the size of individuals caught over time, owing to selection of larger animals and progressive depletion of the largest and oldest. Shifts in size composition can be detected using the physical remains from fishing, such as bones and shells recovered from archaeological sites. For example, size and trophic level of reef fish caught declined over hundreds of years of exploitation in prehistoric island fisheries of the Caribbean [24]. Rick [17] found a roughly 50% decline in the size of red abalone over approximately 7000 years of Holocene exploitation in California Channel Island middens.

One of the central tenets of fisheries management is that by reducing a population’s abundance and shifting its size structure towards smaller individuals, fishing will increase productivity. Smaller individuals grow faster than larger, and animals at lower densities experience less competition for resources. So early human exploitation could have inadvertently produced positive fisheries outcomes from a ‘management’ perspective.

More intense exploitation can push the balance to negativity, however. Fishing and hunting have greater effects on species that are more intrinsically vulnerable. They include animals with life histories such as large body size, late reproduction and low fecundity. Whalers were so ruthlessly efficient that by the early nineteenth century, they had to undertake multiyear expeditions ranging across entire oceans to fill their holds [25]. By the 1830s, newly discovered seal rookeries, such as those of the South Orkney Islands off Antarctica, were stripped in the space of a handful of years [25]. Susceptibility to depletion also extends to animals that are more vulnerable to capture, such as flightless birds, or those which gather at predictable places and times, typically to feed or breed. Such impacts can be detected by earlier declines in the abundance of remains compared to other species. The great auk, once a source of multiple commodities—food, bait, oil and feathers—was driven extinct by 1844, precluding all further use [25].

Impacts on species that are hunted and fished may have led in turn to changes in the environment. For example, analyses of faunal remains from seabed sediment cores from Bocas del Toro in Panama reveal that parrotfish abundance and reef growth rates were positively correlated, mediated by parrotfish grazing promoting growth of coralline algae and coral [26]. Prehistoric declines in parrotfish abundance, which may have been caused in part by human exploitation, led to slower reef growth.

People have been a potent force in reshaping marine ecosystems by other means than direct exploitation. The earliest of these impacts probably arrived as downstream consequences of land use change—owing to the use of fire and land clearance for agriculture—which increased rates of sediment run-off and accumulation [27]. These effects may be accompanied by others, such as nutrification, increased productivity and oxygen depletion. The latter is also a consequence of human and industrial effluent release. Dubois *et al*. [28] reviewed evidence from lake-bed cores for impacts of human land-use change on freshwater aquatic ecosystems. The impacts would have continued downstream into estuarine and open ocean environments, where the signals are harder to detect. The construction of dams later on reduced sediment supplies to coastal waters, but would have impacted coastal ecosystems in other ways by interfering with quantities and annual patterns of run-off.

Depletion and loss would have exacerbated some of these changes in ecosystem functioning. For example, water quality in estuaries would have declined owing to the dual influences of increased river-borne pollution and loss of filter-feeding organisms from direct exploitation and use of destructive fishing methods, such as dredges and trawls [29]. For example, a 600 year timeline based on seagrass sediment cores from Oyster Bay in southwest Australia revealed increased rates of sedimentation from 1850 owing to European settlement and land clearance for agriculture [30]. This preceded a later phase of eutrophication from the 1960s which led to widespread loss of seagrass as water quality declined.

## 5. What can be learned about the past from historical observations?

Historical observations are much maligned as unscientific, selective and perhaps anecdotal. Was an observation only recorded because it was exceptional? Alternatively, was there some motive for exaggeration or misrepresentation such as to encourage new settlers to a territory or attract investment into hunting and fishing expeditions?

However, historical observations can open windows into past worlds that few other sources can. They are often the closest thing we have to eye-witness accounts. Historical accounts often rely on metaphors to convey the sense of a scene. For example, John Mason, Governor of Cupid’s Cove in Newfoundland wrote in 1620 [31], ‘Cods so thicke by the shoare that we heardlie have been able to row a Boate through them, I have killed of them with a Pike’. In Kamchatka, Stepan Krasheninnikov [32] wrote in 1755 of the immense salmon runs there, that ‘the fish come from the sea in such numbers, that they stop the course of the rivers, and cause them to overflow the banks …. They swim up the rivers with such force that the water seems to rise like a wall before them’ (p. 143-145). Both quotes impart a vivid sense of extraordinary abundance that feels like it is based on lived experience rather than conjured by fertile imagination.

Although particular care must be taken to understand their historical context, sometimes observations offer tantalizing glimpses of past environments. The first known report of bottom trawling was a complaint to the English King Edward III in 1376 which described the impact of a trawl as ‘destroying the flowers of the land below water, upon which the great fish are accustomed to be fed’ [25, p. 132]. The phrasing suggests not that the complainants only saw the bycatch of habitat-forming organisms come up in nets, but that they observed those organisms *in situ* on the bottom and were appalled by the swathes that trawls cut through them. This would only be likely in clear shallow water, an impossibility today in muddy estuaries like the Thames where they fished, suggesting very different water quality from now.

**Figure 1.**
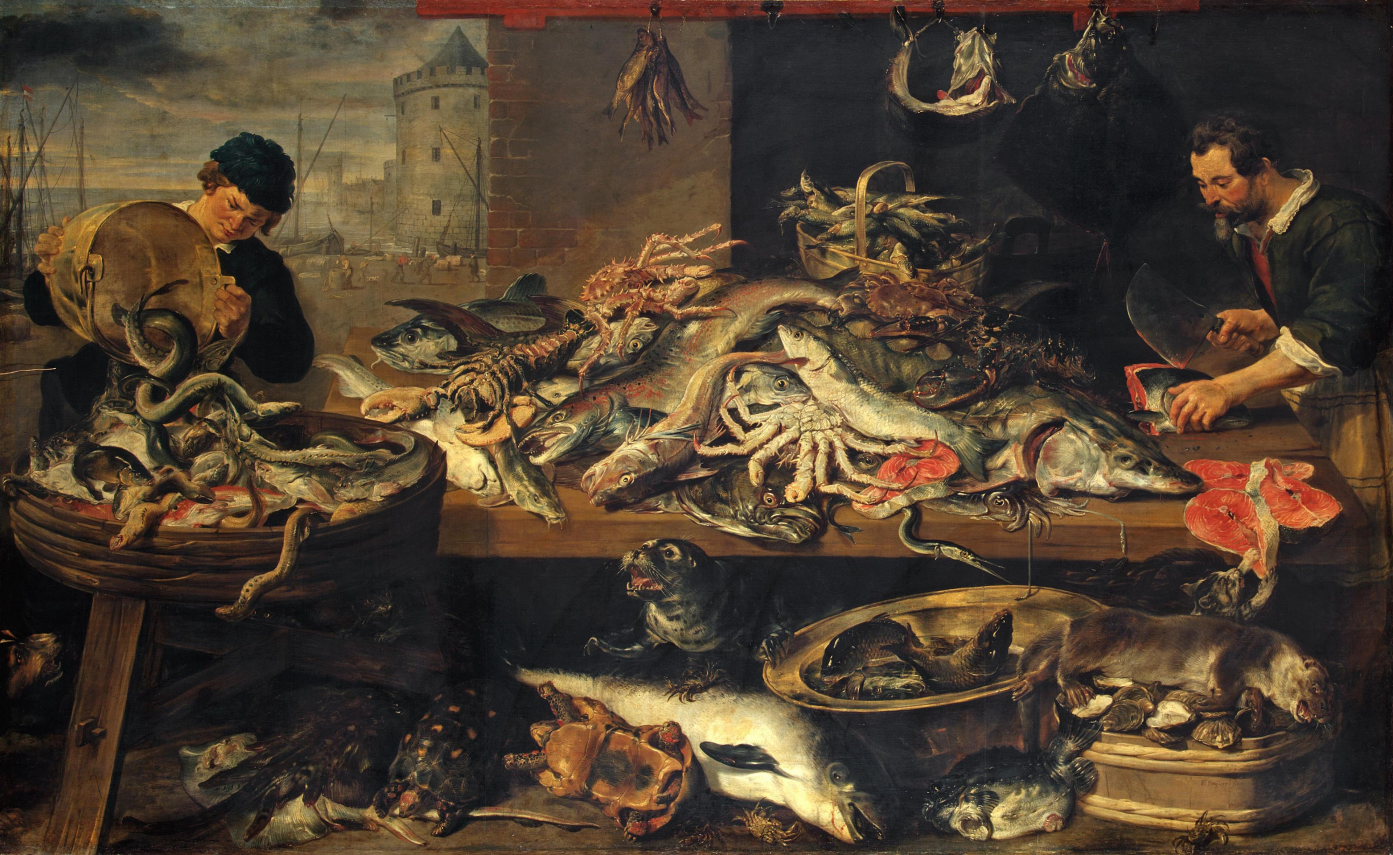
The Fish Stall, by Frans Synders and Jan Wildens, 1618−1621, Hermitage Museum. Old paintings offer windows to the past. Here, although the scene is contrived for artistic effect, the animals depicted are real and accurately portrayed. However, many species shown here are rare or absent from the southern North Sea today, such as sea lamprey, dangling from the basket on the left, the sturgeon and halibut on the table and the oysters at the bottom right. Their presence speaks eloquently to long-term change. The animals are also generally much larger than caught today, but within natural size ranges, like the huge salmon at centre, suggesting the artist had seen them at first hand. There are also animals not eaten today, but consumed at the time, like harbour porpoise and otter, as well as an exotic, the South American red-footed tortoise, recently brought back from the Americas.

Paintings and photographs also offer snapshots of the past. Seventeenth century artists from the low countries of Europe often depicted scenes from food markets. Fran Snyders, a master of the art, painted fish markets on multiple occasions (figure 1). The composition of fish species and their sizes are telling. The fish shown were often giants by the standards of today, including cod and ling over a metre in length, but their sizes lie within the range of biologically possible lengths. The paintings are therefore not crude exaggerations, but suggest that artists populated their pictures with animals they knew to exist because they had seen them. The paintings also included many species rare or absent from local waters today, such as sturgeon, sea lamprey, salmon, halibut and oysters, telling us about the nature of the ecosystems from which they came and how they have changed over subsequent centuries. Similarly, the sea anglers in old photographs and postcards may be mute, but the catches beside them are eloquent envoys for their time, revealing to us, for example, levels of catch per unit effort impossible today, or giant fish that would be record breakers for us.

There is more than one wit who has noted ‘the plural of anecdote is data’. Examples include the use of archives of old photos to conduct rigorous quantitative assessments. Castagnino *et al*. [33] used 275 old photographs to validate local ecological knowledge of changing fish abundance in coastal Peru. Historic photographs also revealed a collapse in the average size of recreational fish landings from Key West between 1956 and 2007 from 19.9 to 2.3 kg [34], and range contraction and altered habitat use by smalltooth sawfish in the United States [35]. Information on species presence, size, catch rates and relative abundance has been extracted from written documents, including government reports, logbooks, magazines and newspapers, and subsequently analysed to infer abundance and/or ecological changes [36, 37,38]. Popular media is a rich but underused source for tracking cultural phenomena such as commercial and recreational fishing activities, and other human activities, over years to decades, sometimes enabling multi-decadal time series to be built [39,40]. Typically, these sources provide contextual information that enables us to posit drivers of observed changes, such as the introduction of new markets or technologies [40,41]. Similarly, oral histories and semi-structured interviews have been used to analyse local and Indigenous ecological knowledges that recollect lived and collectively remembered experiences of environmental change, both within and across generations [42,43].

Historical records do not reach as far back in time as palaeoecological and archaeological data, although some indigenous knowledge is based on oral traditions that can span millennia [44]. Despite a predominantly shorter time frame, written, pictorial and oral accounts cover periods of significant societal and ecological change. Some of these sources can be robustly assessed to identify the magnitude and drivers of ecological change over time periods of decades to centuries. More anecdotal forms of data, while they must be interpreted with care, enable us to contrast past with present experience. They further represent vivid complements to other sources, challenge our established perceptions of the past and help us piece together a more complete picture [45].

## 6. From multiple fragments to the whole

One of the biggest risks in piecing together a history of environment and people is to imprint our own thoughts and prejudices onto the data. There are many possible interpretations for the same data, and no matter how objective we try to be, narrowing them down is partly a matter of subjective choice. In court cases, the protagonists go to great lengths to establish facts, but the defence and prosecution seek to persuade the jury of their different interpretations of what the facts mean. Given that data and interpretations, whether palaeobiological, geological or historical, are vulnerable to subjective preconceptions, the interpretations proposed should be treated as hypotheses open to investigation by scientific means, just like any other hypothesis. While it is true that one cannot replicate the observations of eighteenth century observers, their interpretations can be evaluated against other sources of evidence.

Interpreting a collective of past remains requires the exercise of parsimony. For example, to explain evidence of cannibalism, scientists argued that the Neanderthals were starving because they found it hard to hunt in the rich, deep forests that replaced the glacial steppes, and that there were not enough prey for them [46]. These forests were full of wildlife and Neanderthals were competent and ingenious. That explanation for cannibal practices fails to be supported by limited alternative foraging opportunities [47].

The first step in trying to resolve the issue of cognitive bias in the interpretation of past remains must be to try and fully recognize our biases and attempt to objectively address them in interpretations. Rather than just accepting explanations that easily accord with our own beliefs, we should demand a higher standard of evidential support to make an interpretation that goes along with our cognitive biases rather than challenges them. Another important way to challenge cognitive biases in interpretation is to assemble broadly interdisciplinary teams and to make those teams consist of people who do not always agree. This helps overcome the natural tendency with regard to the evidence and ideas of one’s own discipline as more reliable, or at any rate preferable, to anyone else’s.

Bringing together multiple sources of evidence also helps overcome the limitations of narrow perspectives. Each source and piece of evidence represents a filter through which we see only a fragment of the past, and which may give us biased information. The remains present in middens, for example, are visible through the filter of human preferences and technological capability to access those resources, as well as abundance and differential preservation. An animal might be incredibly abundant but be just beyond the reach of the technologies of the time. Alternatively, an animal might be abundant but shunned because of bad flavour, social taboos or because it was dangerous.

In geology, the study of contemporary processes, such as erosion and sedimentation, produced a great advance for geology in the late eighteenth and early nineteenth centuries, enabling us to better understand the great age of planet Earth. In the biological world, present conditions also offer useful insights, perhaps the most useful of which is that there is a lot of variation in natural communities, only some of which can be explained by their environment. The links between environment and nature are not fully deterministic and are contingent on the species present, which change with time. Today’s ocean productivity might be lower than in the past, for example, not because of altered environmental conditions, but because megafaunal depletion has reduced nutrient supplies to surface waters [48].

Probably the most secure inferences about the past come from the application of multiple sources of evidence simultaneously. Such integration must recognize differences in time scale and time resolution between different sorts of temporal records, whether archaeological, documentary, palaeoecological or the results of direct observation or oral tradition. No one type of record is better or worse than any other. They are different in their different ways and all require critical evaluation through different sorts of procedures, and contribute different perspectives of equal value [49]. Generally, data from the deeper past tend to be of coarser resolution but of greater breadth and time depth, while more recent data are of higher resolution but more narrowly focused and short term. Even taken together, multiple lines of evidence may leave many uncertainties about the environment, nature and people.

## 7. Applying bones and baselines to conservation and management today

Conservation practice often seeks to maintain or recover nature to some ‘ideal’ state. While this ambition should facilitate the integration of historical perspectives, insights from deeper timescales are rarely included in marine conservation and management. This is typically owing to three reasons: first, long-term data often remain unknown to decision makers or have not yet been uncovered by researchers, rendering the data inaccessible [50]; the second is that the patchy nature, varying spatial and temporal resolutions, and uncertainties often inherent in data from the past and its interpretation do not always fit seamlessly into existing management frameworks [38,50]; and third, the use of such data meets resistance owing to a belief that historical perspectives are superfluous in this era of rapid environmental and social-economic changes, where new environmental norms are being established by global trade, invasive species, accelerating climate change and coastal development, among others.

These prevailing notions are, however, being increasingly challenged by practitioners, managers and researchers, who are successfully identifying both the negative implications of ignoring deep time perspectives and the benefits of integrating historical perspectives into conservation and management frameworks, despite the ‘non-analogue’ futures ahead of us [4].

Perhaps the broadest use of historical data has been to challenge prevailing management or assessment paradigms, or their ‘status-quo’ [51]. Researchers have shown how a lack of historical perspective results in conservation or restoration targets that fail to account for past species declines and subsequent changes in ecosystem functioning, such as the previously recounted unrecognized loss of predatory fish on the Dogger Bank [6], and the loss of flat-oyster reefs [9,10]. Once known, these historical data can help establish definitions for reference ecosystems, which in turn aid a more accurate assessment of their current status (e.g. [21]) and present the case for scaling up of restoration efforts [52]. The oft-differing resolution of data from the past, while difficult to integrate with more contemporary information, can also offer up new hypotheses and challenge established scientific norms, such as historical variations in abundance that are not detectable within more truncated time series [53]. Such perspectives can identify whether recent trends in, for example, a stock’s biomass, are within the historical range of variability [54]. While it is often supposed that data from the past are more coarsely resolved than contemporary data, this is not always the case. Indeed, dating techniques such as those used in sclerochronological studies or to date the time of death of coral skeletons [11,55,56], and written archives such as logbook data, newspaper reports and fish trap records, have provided highly temporally and spatially resolved information on species growth rates, distribution, spawning locations, size and/or proxies of abundance [36,53,57]. What may also help embed past perspectives in modern management is increased collaboration between marine historical ecology practitioners and resource managers during data collection and analysis, with the aim to develop datasets that are both intelligible and comparable to contemporary measurements.

Less common are examples where historical data have been directly applied to existing assessment frameworks. Yet collaborations between researchers and managers have successfully integrated such data. Archival and local ecological knowledge data have extended fishery time series landings, catch rate or spatial distribution data [58,59], generated reference points (e.g. population size and age structures) prior to significant human influence [50], and challenged model assumptions such as the timing of introduction of significant fishing pressure, or the role a species played within the ecosystem prior to its decline [50,60]. While information is rare on the degree to which trophic functioning and ecosystem service provision are lost as a species’ role within the ecosystem declines, historical data can underscore where this was likely the case, opening the door to future research questions and opportunities [10].

Historical perspectives can also provide lessons for management and inform conversations and decisions around what societies value and wish to maintain or restore for the future. For example, studies of past Indigenous resource use have provided examples whereby the population age and size structure of a fished species was maintained, despite sometimes intensive exploitation [61]. In Alaska, food web models suggest early Aleuts avoided driving outright loss of prey species by acting as ‘super-generalist and highly omnivorous’ consumers [62]. Robson *et al*. [63] extracted demographic information from over 2000 European oyster (*Ostrea edulis*) shells in Danish shell middens dating from approximately 5660 to 2600 calibrated years BCE. Long-term foraging reduced the age of exploited animals from the Mesolithic (mean: 4.9 years) to the Neolithic (mean: 3.7 years). However, over the full time series, oysters remained a valued and sustainable food source.

In the Venetian Lagoon in the Mediterranean, an early form of fishery co-management was established, which successfully regulated fishing effort there over several centuries [64]. Studies have also shown at what point these relationships broke down and identified the key drivers behind observed shifts, such as the expansion of colonialism [61,65], and the regulatory institutions and circumstances that either promoted or altered exploitative behaviours [64,65]. Studies have, for example, shown instances where decades to centuries of human interactions altered the landscape, but preserved species of contemporary conservation interest or cultural value [66]. Finally, while climate change is often considered as diverging from past conditions, thus rendering past perspectives less useful, as we discover more about how our activities have influenced species distribution, abundance and behaviour over the centuries, areas of convergence are emerging. These include the need to restore ecosystem functioning, enhance connectivity across scales and the necessity of restoration and protection at scale to mitigate cumulative human impacts and enhance species and ecosystems’ resilience to future environmental changes [51,52].

## 8. Conclusions

The study of past oceans and how human influences on them developed and grew over time is entering an exciting new phase. Research interest has expanded rapidly and the investigative toolkit is proliferating across multiple disciplines to constantly rework and refine knowledge. Marine environmental histories can only be constructed by bringing together disparate sources of knowledge, melding the humanities and natural sciences. Studies of particular places and times—postcards from the past—are coalescing into extended vistas as knowledge grows. That research reveals repeated patterns in human interaction with the sea and marine life, and common outcomes. There are still large gaps in understanding, places where our view is partial or obscured. However, our science has firmly rejected the notion that the sea was somehow beyond human influence in the past, and that any impacts were minor and local. Just as on land, people have reshaped marine ecosystems, and did so a surprisingly long time ago. Our newfound knowledge of the past is helping us better understand the present, providing deeper baselines against which to measure change. It is also helping us be more ambitious about the future, setting bolder targets for ecosystem recovery, restoration and rewilding.

## Data Availability

This article has no additional data.

## Authors' Notes

For the purpose of open access, the author has applied a Creative Commons Attribution (CC BY) licence to any Author Accepted Manuscript version arising from this submission.
